# Pancreatic and multiorgan resection with inferior vena cava reconstruction for retroperitoneal leiomyosarcoma

**DOI:** 10.1186/1477-7819-7-3

**Published:** 2009-01-06

**Authors:** John A Stauffer, G Peter Fakhre, Marjorie K Dougherty, Raouf E Nakhleh, William J Maples, Justin H Nguyen

**Affiliations:** 1 Section of General Surgery, Mayo Clinic, Jacksonville, Florida, USA; 2 Division of Transplant Surgery, Mayo Clinic, Jacksonville, Florida, USA; 3 Department of Laboratory Medicine and Pathology, Mayo Clinic, Jacksonville, Florida, USA; 4 Division of Hematology and Oncology (W.J.M.), Mayo Clinic, Jacksonville, Florida, USA

## Abstract

**Background:**

Inferior vena cava (IVC) leiomyosarcoma is a rare tumor of smooth muscle origin. It is often large by the time of diagnosis and may involve adjacent organs. A margin-free resection may be curative, but the resection must involve the tumor en bloc with the affected segment of vena cava and locally involved organs. IVC resection often requires vascular reconstruction, which can be done with prosthetic graft.

**Case presentation:**

We describe a 39-year-old man with an IVC leiomyosarcoma that involved the adrenal gland, distal pancreas, and blood supply to the spleen and left kidney. Tumor excision involved en bloc resection of all involved organs with reimplantation of the right renal vein and reconstruction of the IVC with a polytetrafluoroethylene graft. The patient recovered without renal insufficiency, graft infection, or other complications. Follow-up abdominal imaging at 1 year showed a patent IVC graft and no locally recurrent tumor. Prosthetic graft provides a sufficient diameter and length for replacement conduit in extensive resection of IVC leiomyosarcoma.

**Conclusion:**

To our knowledge, this is the first case of resection of an IVC sarcoma with prosthetic graft reconstruction in combination with pancreatic resection. Aggressive surgical resection including vascular reconstruction is warranted for select IVC tumors to achieve a potentially curative outcome.

## Background

Inferior vena cava (IVC) leiomyosarcomas are rare malignancies; fewer than 300 have been reported in literature. This mesenchymal tumor is derived from medial smooth muscle cells and most often originates from the IVC segment between the hepatic veins and the renal veins [1]. It is most commonly diagnosed in women in their sixth decade, and the tumors often reach large dimensions before detection because of an absence of symptoms [2-6]. They are slow-growing and potentially curable by complete and margin-free resection but are well known to present difficulties in resection because of their location and involvement of surrounding organs and vascular structures.

Locally involved organs are commonly the kidney, adrenal gland, and liver [2,3,5-8]. Radical resection of the tumor en bloc with the affected segment of the vena cava has been shown in multiple studies to be a feasible option with improved survival [1-3,5,6,9,10]. The pancreas is not often involved with this retroperitoneal sarcoma, and pancreas resection may increase the risk of graft infection. Indeed, to our knowledge, a concomitant retroperitoneal sarcoma resection and pancreas resection with IVC interposition grafting have not been reported in literature. We describe a patient with a leiomyosarcoma involving the left kidney, left adrenal gland, and distal pancreas, which required IVC resection followed by reconstruction with polytetrafluoroethylene (PTFE).

## Case presentation

**Figure 1:**
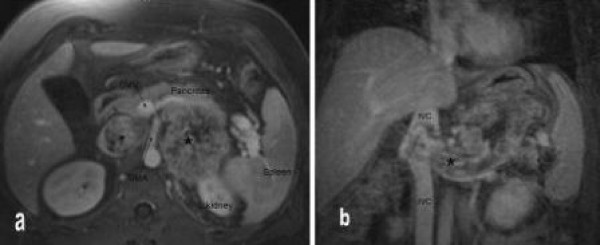
**a, Magnetic resonance imaging shows the tumor (large asterisk) lying to the left of the superior mesenteric artery (SMA), involving the distal pancreas anteriorly, the superior pole of the left kidney posteriorly, and extending into the inferior vena cava (IVC) (small asterisk)**. b, The tumor (large asterisk) involves and encases the left renal vein and extends into the IVC. SMA indicates superior mesenteric artery.

A previously healthy 39-year-old man presented to the emergency department with a 2-month history of intermittent dull abdominal ache with weight gain as well as intermittent right upper extremity numbness. Physical examination revealed a mildly obese abdomen with a subtle mass in the left upper quadrant. No lower extremity edema was noted. Abdominal magnetic resonance imaging revealed a 15 × 6 × 5-cm, well-circumscribed, preaortic retroperitoneal mass, which involved the IVC, causing mass effect on the surrounding organs (Figure 1a, b). The mass was believed to originate from the IVC but was without total IVC occlusion. Tumor involved the left renal artery, splenic artery, and distal pancreas. Hemoglobin, platelet, serum urea nitrogen, creatinine, liver function test, α-fetoprotein, carcinoembryonic antigen, and CA 19-9 findings were all within normal limits. Cells obtained by computer tomographically guided needle biopsy stained positive for vimentin and desmin, confirming the mass was a high-grade retroperitoneal leiomyosarcoma. Further imaging revealed metastatic involvement of the fifth cervical vertebra and epidural membrane. Over the course of the next 8 months, the patient underwent a C5 corpectomy and fusion with removal of the epidural tumor for his metastatic lesion and received 50.4 Gy intensity-modulated radiation therapy to his abdomen, 43.2 Gy to his cervical spine, and 4 cycles of ifosfamide and doxorubicin chemotherapy. Subsequent evaluation showed isolated disease in the retroperitoneum, and the patient was considered to be a candidate for resection with IVC reconstruction of his symptomatic primary tumor.

**Figure 2:**
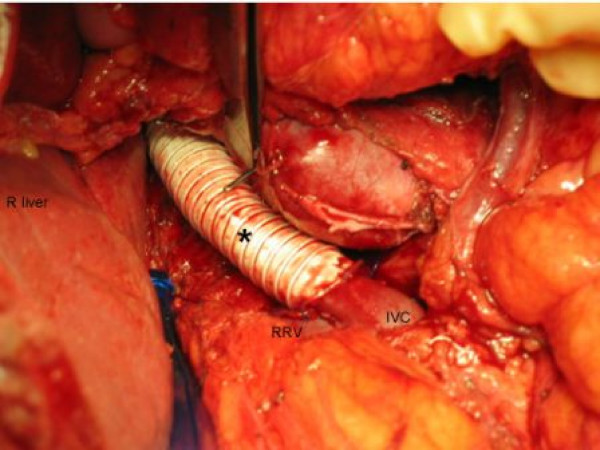
**The interposition polytetrafluoroethylene graft (asterisk) was anastomosed superiorly to the inferior vena cava (IVC) just below the liver, and inferior to the infrarenal IVC, the right renal vein (RRV) was reimplanted into the native IVC**.

**Figure 3:**
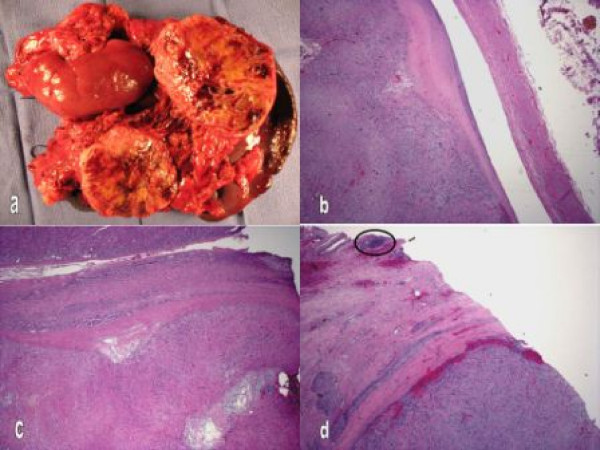
**a, Specimen contained tumor mass, left kidney, left adrenal gland, spleen, and distal pancreas**. b, Leiomyosarcoma is seen in the lumen of the vena cava. The vena cava wall is on the right. c, Tumor fills the bottom of the picture pushing into the adrenal gland seen at the top of the picture. d, Tumor has replaced a portion of the pancreas. A pancreatic islet complex is marked (hematoxylin and eosin, original magnification × 20).

Surgical resection of the mass was performed through a bilateral subcostal incision. Careful abdominal exploration confirmed the preoperative findings, and no other metastases were present. The tumor appeared to arise from the IVC at the level of the left renal vein and extend intraluminally in the IVC up to the caudate lobe, involving the left kidney, left adrenal gland, distal pancreas, splenic artery, and left renal artery. The tumor was mobilized en bloc with the left kidney, left adrenal gland, distal pancreas, and spleen. Proximal and distal control of the IVC was obtained, and the tumor was resected. The pancreas was transected with a linear stapling device, the main pancreatic duct was identified and oversewn, and a closed suction drain was placed at the transection site. Adequate margins were ensured by frozen section. IVC reconstruction was performed from the level of the caudate lobe to the distal IVC in an end-to-end fashion with a 14-mm external ring-reinforced PTFE interposition graft (Figure 2). The graft was wrapped with omentum and isolated from the overlying viscera. The right renal vein was reimplanted into the infrarenal IVC. Gross and histopathologic examination revealed high-grade leiomyosarcoma originating from the IVC involving the adrenal gland and pancreas (Figure 3). After the operation, the patient's renal function remained intact, and he was discharged from the hospital on postoperative day 17 on low-dose oral anticoagulation for 3 months.

**Figure 4:**
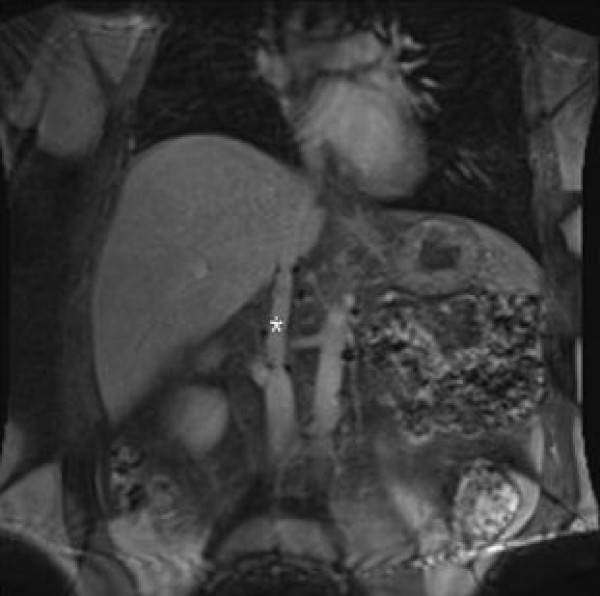
**One-year follow-up magnetic resonance image shows patent polytetrafluoroethylene graft (asterisk) and no local tumor recurrence**.

Postoperatively, the patient underwent adjuvant chemotherapy with 4 cycles of docetaxel and gemcitabine, resection of a metastatic left deltoid tumor mass, and 40.0 Gy of radiation therapy to his left upper extremity. Follow-up abdominal imaging at 1 year revealed no recurrent abdominal disease and a patent IVC graft (Figure 4).

## Discussion

Primary leiomyosarcoma of the IVC is a rare malignant tumor first described in 1891 by Perl at autopsy. The most common presenting symptoms are abdominal pain, palpable abdominal mass, and lower limb edema [1]. However, even with extensive caval involvement, severe venous obstructive symptoms are not often seen, probably because of the development of extensive venous collaterals, which maintain adequate flow around the level of obstruction [2]. The segment of IVC between the renal veins and the hepatic veins (level II or middle segment) is the most commonly affected location for all primary vascular tumors [3,5,6].

IVC leiomyosarcomas are relatively resistant to chemotherapy and radiotherapy, and complete resection of the tumor is the only known method for a chance of cure. The prognosis for leiomyosarcoma of the IVC treated medically is poor, with an average survival of less than 3 months [1]. However, in the past 2 decades, aggressive surgical resection has yielded notable survival benefits, even for patients with metastatic disease. While data are confined to a relatively small number of patients, 5-year survival rates have been shown to be as high as 31% to 53% [3,5-8,10] after complete resection of level II IVC leiomyosarcoma.

Early diagnosis is rare, and the tumors often invade surrounding organs. The amount of vascular involvement by the retroperitoneal tumor accounts for the high surgical risk and technical difficulties seen during attempts at complete resection. Accurate preoperative imaging to determine the extent of the tumor is essential for adequate planning, and magnetic resonance imaging is the preferred modality.

Caval management after IVC resection is controversial. Options include primary repair, autologous patching, ligation, or reconstruction with prosthetic graft. Extensive venous involvement and large tumor size often preclude short segment resection with simple repair or patching. Ligation of the IVC is favored by some and has been shown to be well tolerated and generally safe, especially in those with preoperative IVC thrombosis [1,3]. However, there is a risk of late complications such as pain, swelling, and skin breakdown from severe lower extremity edema. Long-term anticoagulation may be necessary in these patients. Suprarenal IVC tumor involvement treated with IVC ligation can place a patient at serious risk for renal insufficiency. Restoration of flow to the right renal vein by reimplantation (or pelvic kidney autotransplantation) is mandatory to maintain right kidney function, but optional for the left renal vein because of the left kidney's considerable collateral drainage through the adrenal, inferior phrenic, gonadal, and paravertebral vessels [11].

Because of the considerable size of these tumors at diagnosis, wide retroperitoneal dissection is often necessary for complete tumor resection, disrupting the preexisting venous channels. This dissection negates any collateral flow that achieved venous decompression preoperatively. Long segments of tumor involvement of the IVC necessitate ligation of a larger amount of lumbar veins that serve as collaterals. Kieffer et al [5] used a proximal pressure reading of 30 mm Hg or more in the IVC as an indication for caval reconstruction and found reconstruction to be necessary in most cases. PTFE is the most commonly used prosthetic material and has been shown to be a suitable replacement for the IVC with excellent long-term patency [5,6,8-10,12]. Infection and graft thrombosis are the 2 major complications of this type of reconstruction, but both are rare. Graft thrombosis may or may not have any clinical importance, and methods used to decrease its incidence include the use of ring-reinforced PTFE to prevent compression, short-term anticoagulation, and placement of an arteriovenous fistula to augment flow [5].

Although increasing the complexity of the operation, partial or total resection of locally involved organs is necessary for complete tumor removal because prognosis is highly dependent on a tumor-free margin. Patients with inadequate resections are at high risk for local recurrence, causing death from a retroperitoneal sarcoma [3]. Multivisceral resection, especially of enteric organs, may make a surgeon hesitant to place autogenous material for reconstruction. However, PTFE graft infection after IVC replacement has been shown to be a rare occurrence in several large series [5,6,8-10,12]. Measures to decrease risk of graft contamination include routine perioperative intravenous antibiotics, antibiotic irrigation of the abdomen, and coverage of the graft with omentum for graft isolation. However, to our knowledge, resection of the pancreas has not been reported in combination with IVC resection and reconstruction. Pancreatic fistula occurs in up to 23% to 26% of cases of distal pancreatectomy for malignancy [13,14]. Pancreatic leak would have serious consequences in the face of prosthetic vascular material in close proximity and could result in catastrophic graft infection. Measures to prevent pancreatic contamination of the graft should be undertaken, including ensuring adequate distal pancreatic stump closure and providing sufficient closed suction drainage of the pancreatic bed.

## Conclusion

Although often not curative, aggressive surgical resection combined with chemoradiotherapy has been definitively shown to prolong survival in patients with IVC leiomyosarcomas. Vascular reconstruction is often required, and prosthetic replacement of the IVC with PTFE has been shown to be a safe option for retroperitoneal sarcomas. Graft-related complications are low but may be increased by tumor involvement of the pancreas. However, pancreatic involvement did not preclude resection in this case, giving the patient the survival benefit of a margin-free radical en bloc resection.

## Abbreviations

IVC: inferior vena cava; PTFE: polytetrafluoroethylene

## Consent

Written informed consent was obtained from the patient for publication of this case report and any accompanying images. A copy of the written consent is available for review by the Editor-in-Chief of this journal.

## Competing interests

The authors declare that they have no competing interests.

## Authors' contributions

JAS participated in care of the patient and data collection, participated in study design, participated in literature review and manuscript drafting, participated in manuscript writing and revision, and read and approved the final manuscript. GPF participated in care of the patient and data collection, participated in study design, and read and approved the final manuscript. MKD participated in care of the patient and data collection, participated in study design, and read and approved the final manuscript. REN participated in data collection and study design and read and approved the final manuscript. WJM participated in care of the patient and data collection, participated in study design, and read and approved the final manuscript. JHN participated in care of the patient and data collection, participated in study design, participated in manuscript writing and revision, and read and approved the final manuscript. All authors read and approved the final manuscript.
